# Genetic risk factors for late age-related macular degeneration in India

**DOI:** 10.1136/bjophthalmol-2017-311384

**Published:** 2017-12-19

**Authors:** Anand Rajendran, Pankaja Dhoble, Periasamy Sundaresan, Vijayan Saravanan, Praveen Vashist, Dorothea Nitsch, Liam Smeeth, Usha Chakravarthy, Ravilla D Ravindran, Astrid E Fletcher

**Affiliations:** 1 Aravind Eye Hospital, Madurai, Tamil Nadu, India; 2 Aravind Eye Hospital, Pondicherry, Tamil Nadu, India; 3 Department of Genetics, Dr G Venkataswamy Research Institute, Aravind Medical Research Foundation, Madurai, Tamil Nadu, India; 4 Dr Rajendra Prasad Centre for Ophthalmic Sciences, All India Institute of Medical Sciences, New Delhi, India; 5 Faculty of Epidemiology and Population Health, London School of Hygiene & Tropical Medicine, London, UK; 6 Centre for Public Health, Queen’s University, Belfast, UK

**Keywords:** retina, genetics, macula, epidemiology

## Abstract

**Background/Aims:**

There are limited data from India on genetic variants influencing late age-related macular degeneration (AMD). We have previously reported associations from a population-based study in India (the India age-related eye disease study (INDEYE)) of early AMD and single nucleotide polymorphisms (SNPs) in *ARMS2/HTRA1* and no association with *CFH*, *C2* or *CFB*. Late AMD cases were too few for meaningful analyses. We aimed to investigate SNPs for late AMD through case enrichment and extend the loci for early AMD.

**Methods:**

Fundus images of late AMD hospital cases were independently graded by the modified Wisconsin AMD grading scheme. In total 510 cases with late AMD (14 geographic atrophy and 496 neovascular AMD (nvAMD)), 1876 with early AMD and 1176 with no signs of AMD underwent genotyping for selected SNPs. We investigated genotype and per-allele additive associations (OR and 95% CIs) with nvAMD or early AMD. Bonferroni adjusted P values are presented.

**Results:**

We found associations with nvAMD for *CFHY402H* variant (rs1061170) (OR=1.99, 95% CI 1.67 to 2.37, P=10^−6^), *ARMS2* (rs10490924) (OR=2.94, 95% CI 2.45 to 3.52, P=10^−9^), *C2* (rs547154) (OR=0.67, 95% CI 0.53 to 0.85, P=0.01), *ABCA1* (rs1883025) (OR=0.77, 95% CI 0.65 to 0.92, P=0.04) and an SNP near *VEGFA* (rs4711751) (OR=0.64, 95% CI 0.54 to 0.77, P=10^−3^). We found no associations of *TLR3* (rs3775291), *CFD* (rs3826945), *FRK* (rs1999930) or *LIPC* (rs10468017) or *APOE* ε4 alleles with nvAMD or early AMD, nor between early AMD and rs1883025 or rs4711751.

**Conclusions:**

The major genetic determinants of nvAMD risk in India are similar to those in other ancestries, while findings for early AMD suggest potential differences in the pathophysiology of AMD development.

## Introduction

Genetic risk variants for late age-related macular degeneration (AMD) have been identified and further confirmed in genome-wide association studies (GWAS), the majority of which in studies of European ancestry.^1^ There is less information on late AMD genetic risk in India, with most data coming from one patient/control cohort.^2–4^ We have previously reported genetic results from a large population-based study of people aged 60 and over in India (the India age-related eye disease study (INDEYE)) for early AMD with variants in complement factor H (*CFH*), factor B (*CFB*), component 2 (*C2*) and *ARMS2/HTRA1*.^5^ Late AMD cases were too few for meaningful analyses. In the present paper we present results for late AMD based on an enriched sample and for other genetic loci with early AMD.

## Materials and methods

INDEYE was conducted between 2005 and 2007 in two locations in south (Tamil Nadu) and north (Haryana) India. The study methods including sampling and recruitment, blood collection, ophthalmological examination and AMD grading, along with results on the prevalence of early and late AMD, have been published.^6^ In the present study, we recruited additional cases of late AMD between 2009 and 2011 from the hospitals that participated in the INDEYE study (All India Institute of Medical Sciences, Delhi, and Aravind Eye Hospital, Pondicherry, Tamil Nadu) and additionally from Aravind Eye Hospital, Madurai, Tamil Nadu. We aimed to achieve 600 late AMD cases plus two population controls per case to detect the twofold per-allele association of *Y402H CFH* (rs1061170) reported in a meta-analysis of primarily European ancestry^7^ at 90% power and alpha <0.001. Initial eligibility criteria were age 60 years and over, Indian descent and a diagnosis of late AMD by retinal ophthalmologists. Controls were participants in the INDEYE study with no signs of early or late AMD in either eye.

In both INDEYE and clinic participants, informed written consent was obtained prior to enrolment. If the participant was illiterate, the information sheet was read out aloud in the presence of a local witness, and a thumb impression of the participant signified assent. The study complied with the Declaration of Helsinki.

Full details of the method of ascertainment of AMD in the population study have previously been published.^6^ In brief two 35° stereo fundus photographs of each eye were taken and graded at Queens University Belfast (QUB) using the modified Wisconsin Age-Related Maculopathy Grading System.^8^ Each eye was classified into four mutually exclusive grades: grade 1: soft distinct drusen (≥63 µm) only *or* pigmentary irregularities only; grade 2: soft indistinct (≥125 µm) or reticular drusen only *or* soft distinct drusen (≥63 µm) with pigmentary irregularities; grade 3: soft indistinct (≥125 µm) or reticular drusen with pigmentary irregularities; grade 4: either neovascular AMD (nvAMD; presence of any of the following: serous or haemorrhagic retinal or retinal pigment epithelial detachment, subretinal neovascular membrane, periretinal fibrous scar) or geographic atrophy (GA; well-demarcated area of retinal pigment atrophy with visible choroidal vessels). Fundus images of cases recruited from hospital clinics were sent to QUB (colour photographs, optical coherence tomography (OCT)) and graded as above. In all graded images, GA and nvAMD present in the same eye were categorised as nvAMD. Images that showed no signs of any features of early or late AMD were categorised as having no AMD.

### DNA extraction and genotyping

Genomic DNA was extracted from peripheral blood leucocytes using Qiagen kits. Single nucleotide polymorphisms (SNPs) were genotyped using TaqMan assays in an ABI 7900 real-time PCR. We limited our study to genes in biological pathways relevant to AMD pathogenesis, including complement activation (*CFH*, *CFB*, *CFD*) and deposition (Toll-like receptors (*TLR 3*, *4*, *7*)), lipid metabolism (*ABCA1*, *APOE*, *CETP*, *LIPC*), or the degradation of the extracellular matrix (*TIMP3*).^9^ We investigated two SNPs on chromosome 6, previously reported to be associated with late AMD^10^ (*LOC107986598* rs4711751 located near *VEGFA* and *FRK* rs1999930 near *COL10A1*). We included SNPs in *ARMS2/HTRA1* due to their demonstrated importance in many studies^11^ and recent evidence for an *ARMS2* role in surface complement regulation.^12^ We tested for departures from Hardy-Weinberg equilibrium (HWE) in controls and excluded any SNPs with a P value ≤0.05. We used logistic regression in Stata V.14 to examine associations of (1) genotype and (2) per-allele additive models adjusted for age, sex and centre. We present additionally Bonferroni-adjusted P values for the number of independent SNPs tested. We created *APOE* alleles from the SNPs rs429358 (T/C) and rs7412 (C/T), resulting in three alleles: ε2 (TT), ε3 (TC) and ε4 (CC). Analyses of *APOE* alleles used ε3 as the reference group.

## Results

**Figure 1:**
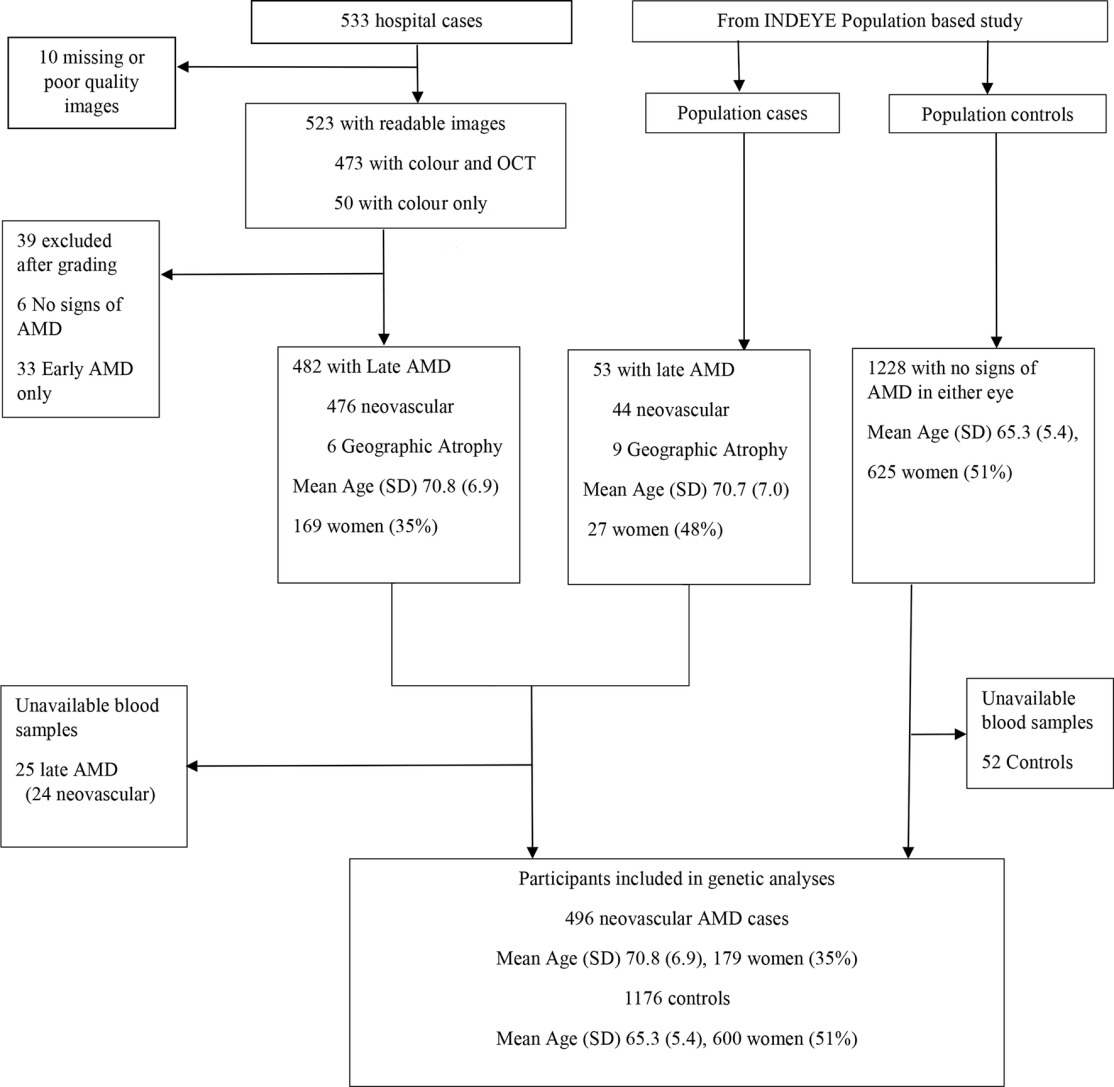
Flow chart of hospital case recruitment and population cases and controls. AMD, age-related macular degeneration; OCT, optical coherence tomography.

The prevalence of early and late AMD in the INDEYE population study has been published.^6^ There were 1986 cases of early AMD (1686 grade 1, 289 grade 2, 11 grade 3), 53 of late AMD (44 nvAMD, 9 GA) and 1228 population controls with no signs of AMD in either eye. Hospital retinal clinics recruited 533 cases based on ophthalmologists’ diagnoses. After exclusion of participants without confirmed late AMD or missing blood samples (figure 1), 496 nvAMD cases, 1876 early AMD and 1176 controls were available for analysis. We did not investigate GA because of a small number (n=14). The mean age in years (SD) was 65.3 (5.4) in population controls, in early AMD 67.0 (6.1) and in nvAMD 70.7 (6.9). The number and proportion of women were 600 (51%), 915 (49%) and 179 (36%), respectively. Two SNPs (rs4986790 *TLR4/TLR7*, rs9621532 *TIMP3*) failed HWE. HWE and minor allele frequencies (MAFs) for the remaining SNPs are shown in table 1. We also present MAFs for European and Indian ancestries from the 1000 genome study (https://www.ncbi.nlm.nih.gov/snp, accessed 5 December 2016). The control frequencies of *APOE* alleles were ε3 (0.73), ε2 (0.09) and ε4 (0.18).

**Table 1:** SNPs, MAF and test for HWE and corresponding reported MAF in the 1000 genomes project in South Asian and European populations

Chromosome	Gene	SNP	Major/minor alleles	HWE*	MAF†	MAF EUR‡	MAF SAS§
1	*Y402H*	rs1061170	T/C	0.6854	0.323	0.362	0.287
4	TLR3	rs3775291	C/T	0.9347	0.235	0.324	0.263
6	*C2*	rs547154	C/A	0.3813	0.187	0.089	0.156
6	*SKIV2L*	rs438999	A/G	0.6932	0.183	0.089	0.148
6	*LOC107986598*¶	rs4711751	T/C	1	0.423	0.487	0.330
6	*FRK*	rs1999930	C/T	0.3957	0.075	0.281	0.052
9	*ABCA1*	rs1883025	C/T	0.0797	0.432	0.240	0.413
10	*ARMS2*	rs10490923	G/A	0.7299	0.149	0.130	0.148
10	*ARMS2*	rs10490924	G/T	0.1953	0.319	0.195	0.343
10	*HTRA1*	rs2672598	T/C**	0.2969	0.524	0.499	0.464
15	*LIPC*	rs10468017	C/T	0.9195	0.176	0.283	0.184
16	*CETP*	rs3764261	C/A	0.0737	0.295	0.292	0.321
19	*APOE*	rs429358	T/C	1	0.097	0.155	0.087
19	*APOE*	rs7412	C/T	0.5232	0.050	0.063	0.044
19	*CFD*	rs3826945	T/C	0.8424	0.344	0.313	0.334

*P value for tests for departure from Hardy-Weinberg equilibrium (HWE) in controls.

†Minor allele frequency (MAF) in controls.

‡MAF from 1000 genome study for European ancestry available at https://www.ncbi.nlm.nih.gov/snp.

§MAF from 1000 genome study for South Asian ancestry available at https://www.ncbi.nlm.nih.gov/snp.

¶SNP located near *VEGFA*.

**Minor allele considered as C for comparison with other studies.

SNP, single nucleotide polymorphisms.

We found additive associations with nvAMD for *Y402H* (rs1061170), *HTRA1* (rs2672598), *ARMS2* (rs10490924, rs10490923), *CFB* (rs438999, rs547154), *ABCA1* (rs1883025) and SNP (rs4711751 close to *VEGFA*) (table 2). We found no associations with *TLR3* (rs3775291), *CFD* (rs3826945), *FRK* (rs1999930) or *LIPC* (rs10468017). There was no association between *APOE* ε4 and nvAMD (OR=0.72, 95% CI 0.52 to 1.01).

**Table 2:** Association of neovascular age-related macular degeneration with SNPs

Gene	SNP	Major/minor alleles	1 vs 0 copy of minor allele		2 vs 0 copies of minor allele		Additive per minor allele			
			OR*	95% CI	OR*	95% CI	OR*	95% CI	P	P†
*Y402H*	rs1061170	T/C	1.72	1.31 to 2.28	4.13	2.91 to 5.87	1.99	1.67 to 2.37	10^−7^	10^−6^
*TLR3*	rs3775291	C/T	1.18	0.92 to 1.51	0.88	0.51 to 1.53	1.06	0.87 to 1.30	0.545	
*C2*	rs547154	C/A	0.62	0.47 to 0. 82	0. 64	0.29 to 1.43	0.67	0.53 to 0. 85	0.001	0.01
*SKIV2L*	rs438999	A/G	0.63	0.47 to 0.83	0.50	0.21 to 1.22	0.65	0.50 to 0.83	0.001	0.01
*LOC107986598*	rs4711751	T/C	0.35	0.27 to 0.46	0.65	0.45 to 0. 94	0.64	0.54 to 0. 77	10^−4^	10^−3^
*FRK*	rs1999930	C/T	0.93	0.64 to 1.34	5.94	1.17 to 30.10	1.05	0.74 to 1.49	0.777	
*ABCA1*	rs1883025	C/T	0.81	0.61 to 1.07	0.58	0.41 to 0.83	0.77	0.65 to 0.92	0.003	0.04
*ARMS2*	rs10490923	G/A	0.49	0.35 to 0.67	0.85	0.33 to 2.17	0.57	0.43 to 0.75	10^−3^	0.04
*ARMS2*	rs10490924	G/T	1.86	1.37 to 2.51	8.73	6.11 to 12.48	2.94	2.45 to 3.52	10^−10^	10^−9^
*HTRA1*	rs2672598	T/C	1.53	1.01 to 2.32	5.42	3.58 to 8.21	2.67	2.19 to 3.25	10^−9^	10^−8^
*LIPC*	rs10468017	C/T	1.13	0.87 to 1.47	1.12	0.56 to 2.23	1.11	0.89 to 1.37	0.370	
*CETP*	rs3764261	C/A	1.27	0.98 to 1.64	1.26	0.82 to 1.91	1.17	0.98 to 1.41	0.087	
*APOE*	rs429358	T/C	0.82	0.60 to 1.14	NC‡		NC‡			
*APOE*	rs7412	C/T	0.87	0.58 to 1.32	NC‡		NC‡			
*CFD*	rs3826945	T/C	1.02	0.79 to 1.31	1.05	0.70 to 1.58	1.02	0.85 to 1.23	0.820	

*Adjusted for age, sex and centre.

†Bonferroni-adjusted P value for 13 per-allele tests.

‡Not calculated, no cases with 2 copies of minor allele.

SNP, single nucleotide polymorphisms.

We combined grades 2 and 3 of early AMD due to the small numbers of grade 3. Subsequently we combined all grades of early AMD (1–3) because our preliminary analyses revealed no differences in genetic associations for these early stages. There were no associations with early AMD and any of the SNPs (table 3) or with *APOE* ε4 (OR=0.88, 95% CI 0.73 to 1.01).

**Table 3:** Association of early age-related macular degeneration with SNPs

Gene	SNP	1 vs 0 copy of minor allele			2 vs 0 copies of minor allele			Additive per allele		
		OR*	95% CI	P	OR*	95% CI	P	OR*	95% CI	P
*TLR3*	rs3775291	1.01	0.86 to 1.20	0.868	1.13	0.83 to 1.54	0.425	1.04	0.92 to 1.16	0.520
*LOC107986598*	rs4711751	0.95	0.78 to 1.14	0.558	0.91	0.69 to 1.20	0.502	0.95	0.84 to 1.08	0.452
*FRK*	rs1999930	0.83	0.66 to 1.05	0.117	1.63	0.39 to 6.77	0.498	0.88	0.69 to 1.12	0.285
*ABCA1*	rs1883025	0.96	0.79 to 1.17	0.698	0.96	0.77 to 1.19	0.726	0.98	0.88 to 1.09	0.699
*LIPC*	rs10468017	0.97	0.82 to 1.14	0.677	1.08	0.67 to 1.75	0.744	0.99	0.85 to 1.15	0.913
*CETP*	rs3764261	1.08	0.91 to 1.28	0.365	1.10	0.90 to 1.33	0.339	1.06	0.96 to 1.17	0.228
*APOE*	rs429358	0.93	0.77 to 1.12	0.454	0.80	0.35 to 1.84	0.594	0.93	0.77 to 1.10	0.380
*APOE*	rs7412	0.97	0.74 to 1.27	0.826	1.38	0.42 to 4.55	0.594	1.00	0.79 to 1.27	0.994
*CFD*	rs3826945	1.02	0.88 to 1.20	0.758	1.10	0.87 to 1.39	0.399	1.04	0.94 to 1.15	0.416

*Adjusted for age, sex and centre.

SNP, single nucleotide polymorphism.

## Discussion

*CFH* and *ARMS2/HTRA1* have been identified in numerous studies in European^1 11^ and East Asian ancestries^13^ as the most important genes for late AMD risk, with effect sizes around 2.5 and 3 per allele, respectively,^1 7 11^ and the top two variants at GWAS significance.^1^ Our effect sizes of 2 for the C allele of *Y402H* variant of *CFH* (rs1061170) and 3 for *ARMS2* T allele (rs10490924) are consistent with these findings and add to the limited evidence for India.^2 3^ The MAF of rs1061170 is lower in East Asian (<0.10) compared with European ancestry (0.3),^7^ and higher for rs10490924 (0.4), almost twice that in European ancestry.^9^ Our MAFs for rs1061170 (0.32) and rs10490924 (0.32) concur with those for South Asians in the 1000 genome study (table 1) and other sources in India.^2 3 14^ It appears that rs1061170 allele frequencies in Indian ancestry are closer to European than East Asian and intermediate between European and East Asian for rs10490924.

We found associations with SNPs in other genes established predominantly in European ancestry, including *C2*, *SKIV2L* and *ABCA1* and in an SNP (rs4711751) in an uncharacterised gene *LOC107986598* close to *VEGFA*.^1^ We found a reduced risk with the T allele of *ABCA*1 (rs1883025) but not with *CETP* or *LIPC*. A meta-analysis of European ancestry studies found *APOE* ε4 haplotype was associated with a 30% lower risk of nvAMD^15^; we observed a similar effect but with wide CIs.

We found no association with early AMD and any of the variants reported in table 3. We have previously reported results for early AMD and found no association with *Y402H* (rs1061170), *C2* (rs547154) and *SKIVL* (rs43899).^5^ *ARMS2/HTRA1* variants (rs10490924 and rs2672598) were associated with early AMD; the OR per allele was 1.22 (95% CI 1.13 to 1.33, P<0.0001) and 1.12 (95% CI 1.02 to 1.23, P=0.02), respectively.^5^ A GWAS meta-analysis of 4089 early AMD cases, the majority of European ancestry, found associations between SNPs in *CFH* and *ARMS2/HTRA1*, but with smaller effect sizes than those reported for late AMD.^16^ Analyses by Asian ancestry found no association with any *CFH* SNP, whereas *ARMS2* (rs10490924) was associated with an OR of 1.18 (95% CI 1.07 to 1.13), similar to our study, compared with 1.43 (95% CI 1.34 to 1.54) for European ancestry. The lower prevalence of early AMD in Asia^17^ and India^6^ may, in part, be explained by the apparently lesser role of genetic variants compared with studies in European ancestry, but caution is warranted due to the paucity of genetic studies of early AMD in Indian and East Asian ancestries.

### Limitations

Although we did not attain the 600 planned cases, we confirmed the per-allele twofold risk of rs1061170 and nvAMD hypothesised for the sample size estimates. We had low power to investigate variants with low MAFs (compared with European ancestry) such as *FRK* and *LIPC*, or to identify smaller effects. The majority of late AMD cases were of nvAMD phenotype, similar to studies in East Asia,^18^ and we could not investigate genetic associations with GA. It is possible we misclassified population cases of late AMD. We had confirmatory OCTs in 89% of clinical late AMD cases, but the population-based study used colour images only.

## Conclusions

Our findings suggest the major genetic determinants of nvAMD risk in India are similar to those in other populations, while findings for early AMD suggest potential differences in the pathophysiology of AMD development.
